# Novel screen for anti-cancer drugs that elevate chromosome instability (CIN) using human artificial chromosome (HAC)

**DOI:** 10.18632/oncotarget.26406

**Published:** 2018-12-07

**Authors:** Natalay Kouprina, Yves Pommier, Vladimir Larionov

**Affiliations:** ^1^ Developmental Therapeutics Branch and Laboratory of Molecular Pharmacology, Center for Cancer Research, National Cancer Institute, NIH, Bethesda, MD 20892, USA

**Keywords:** chromosome instability, CIN, human artificial chromosome, HAC, anti-cancer drugs

## Abstract

Human artificial chromosomes (HACs) bearing functional kinetochores have been exploited as promising systems for gene delivery and expression and in studies of different epigenetic modifications on kinetochore structure and function. The HAC-based technology has been also used to develop drug screening and assessment strategies to manipulate the CIN (chromosome instability) phenotype in cancer cells. More recently, we designed a new protocol for systematic analysis of compounds specifically targeting telomeres and telomerase. This approach used two isogenic cell lines containing a circular HAC (lacking telomeres) and a linear HAC (containing telomeres): compounds that target telomerase or telomeres should preferentially induce loss of the linear HAC but not the circular HAC. This platform enables identification and ranking of compounds that greatly increase chromosome mis-segregation rates as a result of telomere dysfunction and may expedite the development of new therapeutic strategies for cancer treatment.

Aneuploidy is a feature of most cancer cells and is often accompanied by an elevated rate of chromosome mis-segregation termed chromosome instability (CIN). While CIN can act as a driver of cancer genome evolution and tumor progression, recent findings point to the existence of a threshold level beyond which CIN becomes a barrier to tumor growth and therefore can be exploited therapeutically [1]. Drugs known to increase CIN beyond the therapeutic threshold are currently few in number and the clinical promise of targeting the CIN phenotype warrants new screening efforts. However, none of the existing methods to quantify CIN is entirely satisfactory [2, 3, 4].

We have developed a new high-throughput assay for measuring CIN. This quantitative assay for chromosome mis-segregation is based on a non-essential human artificial chromosomes (HAC) with a functional kinetochore constructed in our labs for gene delivery [5]. To adopt this HAC for CIN studies, the *EGFP* transgene was inserted into the HAC [6]. Thus, cells that inherit the HAC fluoresce green while cells that lack the HAC do not. This allows the measure of HAC loss rate by routine flow cytometry. In the constitutive genome, it would be extremely difficult to detect significant increases in non-disjunction, as chromosome segregation is very accurate and the number of cells lacking GFP expression would be extremely low. However, the HAC, although it does segregate normally in most divisions, offers a sensitized system with a higher loss rate that is much more readily measured by flow cytometry, particularly after drug treatment. Using this HAC-based assay, we have analyzed a set of well-known drugs with a different mechanism of action, all of which had been reported to induce CIN [7]. The highest rate of HAC mis-segregation was observed for microtubule-stabilizing drugs (Taxol, Dactylolide), inhibitors of Polo-like and Aurora kinases (GW843682 and VX-680), poly(ADPribose)polymerase (PARP) inhibitors (Olaparib, and Talazoparib [BMN-673]), inhibitor of topoisomerase I (TOP1) [Indotecan (LMP400)] developed in our laboratory, inhibitor of DNA synthesis (Gemcitabine), and DNA crosslinking agent (Cisplatin). These compounds may be recommended as the first choice when CIN is considered as a target for cancer therapy. Combination of drugs with different mechanisms of action resulting in chromosome destabilization also may be considered for new clinical trials. It is important that this new and simple assay allows a quick and efficient screen of hundreds of drugs to identify those affecting chromosome mis-segregation [8].

Targeting telomerase and telomere maintenance mechanisms represents a promising therapeutic approach for various types of cancer [9]. In our recent work [10], we modified our HAC-based protocol [6, 7] to screen for and rank the efficacy of compounds specifically targeting telomeres and telomerase. The protocol is based on the use of two isogenic cell lines containing a circular HAC (lacking telomeres) and a linear HAC (containing telomeres) marked with the *EGFP* transgene. Compounds that target telomerase or telomeres should preferentially induce loss of the linear HAC but not of the circular HAC (Figure 1). We applied this novel dual-HAC assay to rank a set of known and newly developed compounds, including G-quadruplex (G4) ligands. Among the latter group, we found two compounds – Cu-ttpy and Pt-ttpy - that induce a high rate of linear HAC loss with no significant effect on circular HAC. Analysis of the mitotic phenotypes induced by these drugs revealed an elevated rate of chromatin bridges in late mitosis and cytokinesis as well as Ultrafine Bridges. Further cytological analysis showed that chromosome loss after Pt-ttpy or Cu-ttpy treatment correlated with the induction of telomere-associated DNA damage [10]. Identification and ranking of compounds that greatly increase chromosome mis-segregation rates as a result of telomere dysfunction may expedite the development of new therapeutic strategies for cancer treatment.

**Figure 1:**
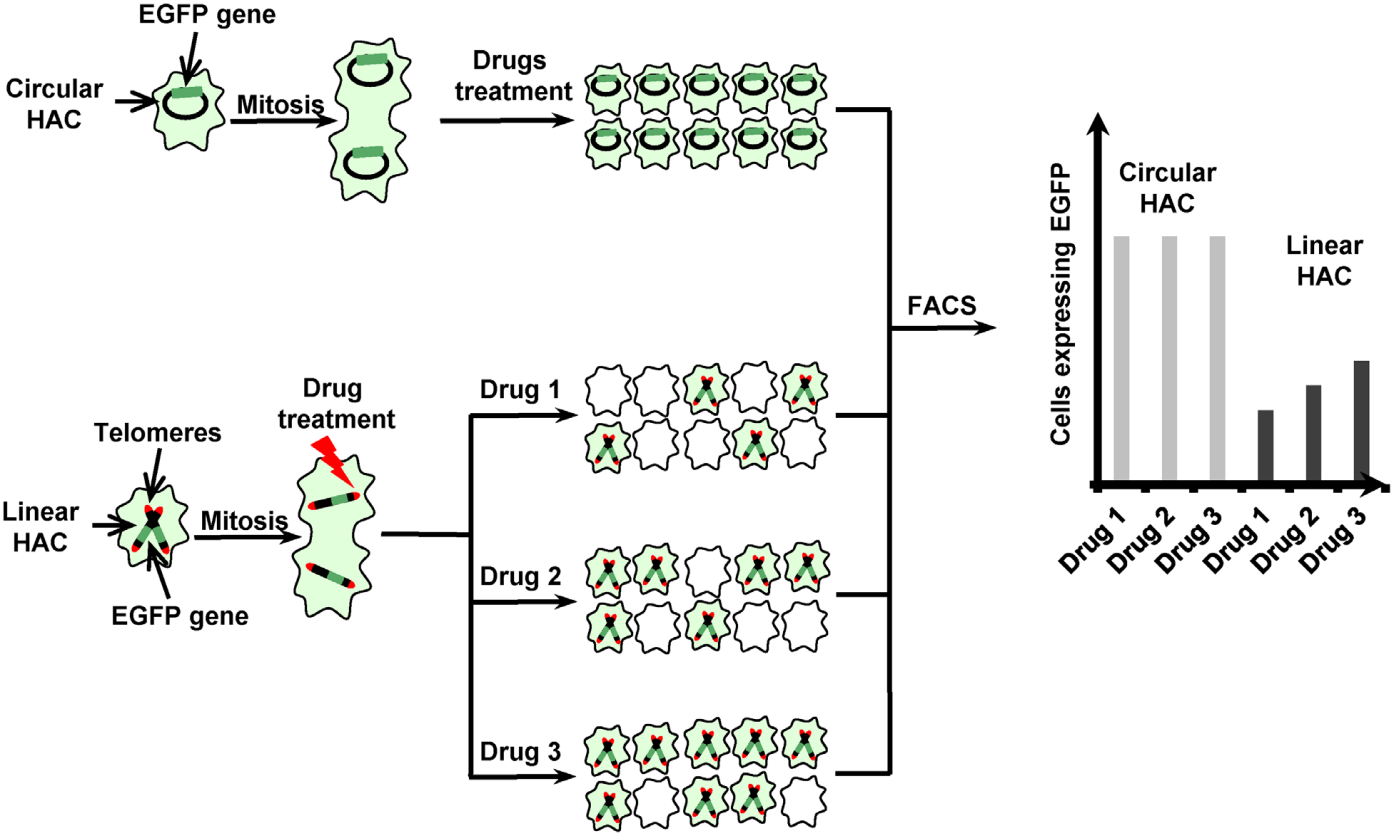
Scheme of an assay for detection of drugs specifically targeting telomerase or telomeres based on the use of linear versus circular HACs, both containing the *EGFP* transgene Cells that inherit any of these HACs display green fluorescence, while cells that lack them do not. Both HACs are stable during cell division. So, the untreated cells and the cells containing a circular HAC display uniform green fluorescence while the cells containing a linear HAC after treatment of drugs that affect telomerase or telomers are highly variable in fluorescence. The actual number of cells with a EGFP-HAC can be measured by FACS as described [10]. Thus, the compounds that increase a linear HAC loss but has no effect on a circular HAC may be identified.

In perspective, a new assay based on a sensitized system to detect chromosome mis-segregation will allow developing straightforward, quantitative assessment of CIN under a variety of conditions. This assay may be employed to identify new compounds/drugs that specifically elevate chromosome mis-segregation and drive lethal aneuploidy. These drugs will lay the foundation for new treatment strategies for cancer.
